# Clinical Investigation of Patients With Anchor Screws and Plates at Tokyo Dental College Chiba Dental Center

**DOI:** 10.7759/cureus.80173

**Published:** 2025-03-06

**Authors:** Mizuki Nakano, Chie Tachiki, Hiromi Tomaru, Yuichiro Osawa, Kentaro Ota, Yuki Iijima, Dai Ariizumi, Akira Watanabe, Akira Katakura, Yasushi Nishii

**Affiliations:** 1 Department of Orthodontics, Tokyo Dental College, Tokyo, JPN; 2 Department of Oral and Maxillofacial Surgery, Tokyo Dental College, Tokyo, JPN; 3 Department of Oral Pathobiological Science and Surgery, Tokyo Dental College, Tokyo, JPN

**Keywords:** anchorage, anchor screw, clinical data analysis, clinical investigation, orthodontics, statistical analysis, temporary anchorage devices (tads)

## Abstract

Purpose

A clinical statistical survey was conducted on the usage of anchor screws and plates over a seven-year period from 2014 to 2021, with the objective of examining trends in their application by comparing the findings with the previous research.

Materials and methods

The subjects of this study were all patients who started comprehensive orthodontic treatment and underwent the placement of anchor screws and plates at the Department of Orthodontics, Tokyo Dental College Chiba Dental Center (formerly Chiba Hospital) between April 2014 and March 2021. The survey collected data on gender, age at the initial exam, type of treatment (self-funded or public medical insurance-covered), patient category, malocclusion classification, and treatment details from medical records.

Results

A total of 655 patients were included, consisting of 197 males and 458 females, with the majority being in their teens and 20s. Of this, 409 patients received implants in self-funded treatments, while 246 received implants in public medical insurance-covered treatment, including 193 patients with jaw deformities. The primary purpose of usage in the maxilla was for maximum anchorage, followed by distal movement. In the mandible, maximum anchorage accounted for 77 (44%), while distal movement comprised 68 (39%).

Conclusion

Our department has also previously reported the clinical statistics of anchor screw and plate usage, where the primary use of anchor screws in the mandible was for distal movement; however, in the current report, the predominant usage shifted to maximum anchorage. Given that further changes in the purposes of anchor screw usage may arise, continuous clinical statistical analysis will be necessary for future observations.

## Introduction

Anchorage in orthodontic clinical practice is defined as a source of resistance to prevent undesirable tooth movement. Various methods have been used to enhance anchorage, including intraoral, intermaxillary, and extraoral anchorage [1,2]. However, intraoral anchorage is less effective compared to other methods and may lead to a deterioration in oral hygiene. Additionally, intermaxillary and extraoral anchorage depend on patient cooperation, making it challenging to achieve consistent treatment outcomes [3]. To secure an absolute source of anchorage in orthodontic treatment, the use of orthodontic anchor screws (hereafter referred to as "anchor screws") and anchorage plates (hereafter referred to as "plates) began, allowing for previously challenging tooth movement patterns.

In the 1980s, Creekmore and Eklund [4] published a case report on using screws as a source of anchorage for tooth movement, and Roberts et al. [5] reported a method of using dental implants placed in the posterior mandible as an absolute source of anchorage for mesial molar movement. In Japan, Kanomi [6] was the first to report on this, and since then, numerous basic and clinical studies on anchor screws have been conducted [7-10]. Our department has also previously reported the clinical statistics of anchor screw and plate usage from 2004 to 2013, presented as the first report [11]. The use of anchor screws and plates has expanded the scope of treatment planning, and since pharmaceutical approval in 2012, various mechanics have been devised. In a report by Masuyama et al. [12], the usage status of anchor screws over five years was discussed. However, there are few reports detailing long-term usage trends, changes following public medical insurance coverage, and the specific purposes of usage. Therefore, we conducted a clinical statistical survey on the usage status of anchor screws and plates in our department over a seven-year period from 2014 to 2021, aiming to compare it to the first report and to examine trends in their application.

## Materials and methods

The subjects of this study were all patients who started comprehensive orthodontic treatment and underwent the placement of anchor screws and plates at the Department of Orthodontics, Tokyo Dental College Chiba Dental Center (formerly Chiba Hospital) between April 2014 and March 2021. Orthodontic patients without anchor screw or plate implantation were excluded in this study. In all cases, diagnoses were conducted by board-certified or clinical instructor orthodontists certified by the Japan Orthodontic Society, who determined the sites, number, and mechanics of anchor screw and plate placement.

The survey items included gender, age at the initial examination, breakdown by self-funded or public medical insurance-covered treatment, breakdown of insurance patients (patients with jaw deformities, cleft lip and palate patients, and patients with other congenital anomalies), classification of malocclusion according to Angle, and treatment details such as the presence or absence of tooth extraction, type of anchor screw and plate, number of placements, placement site, and purpose of use. These data were collected from medical records.

Additionally, a cross-tabulation was performed to examine the relationship between Angle's classification at the initial examination and the purpose of using anchor screws and plates, with a chi-square test conducted at a significance level of 5%. When significant differences were observed in the chi-square test results, a residual analysis was conducted to identify the specific items contributing to the significance. In the residual analysis, items with an adjusted residual absolute value of 1.96 or higher were considered notable.

All analyses were performed using IBM SPSS Statistics for Windows, Version 27 (Released 2020; IBM Corp., Armonk, New York). This study was approved by the Ethics Committee of Tokyo Dental College (approval number 1066).

## Results

Gender, age, and yearly trends in anchor screw and plate placement

**Figure 1:**
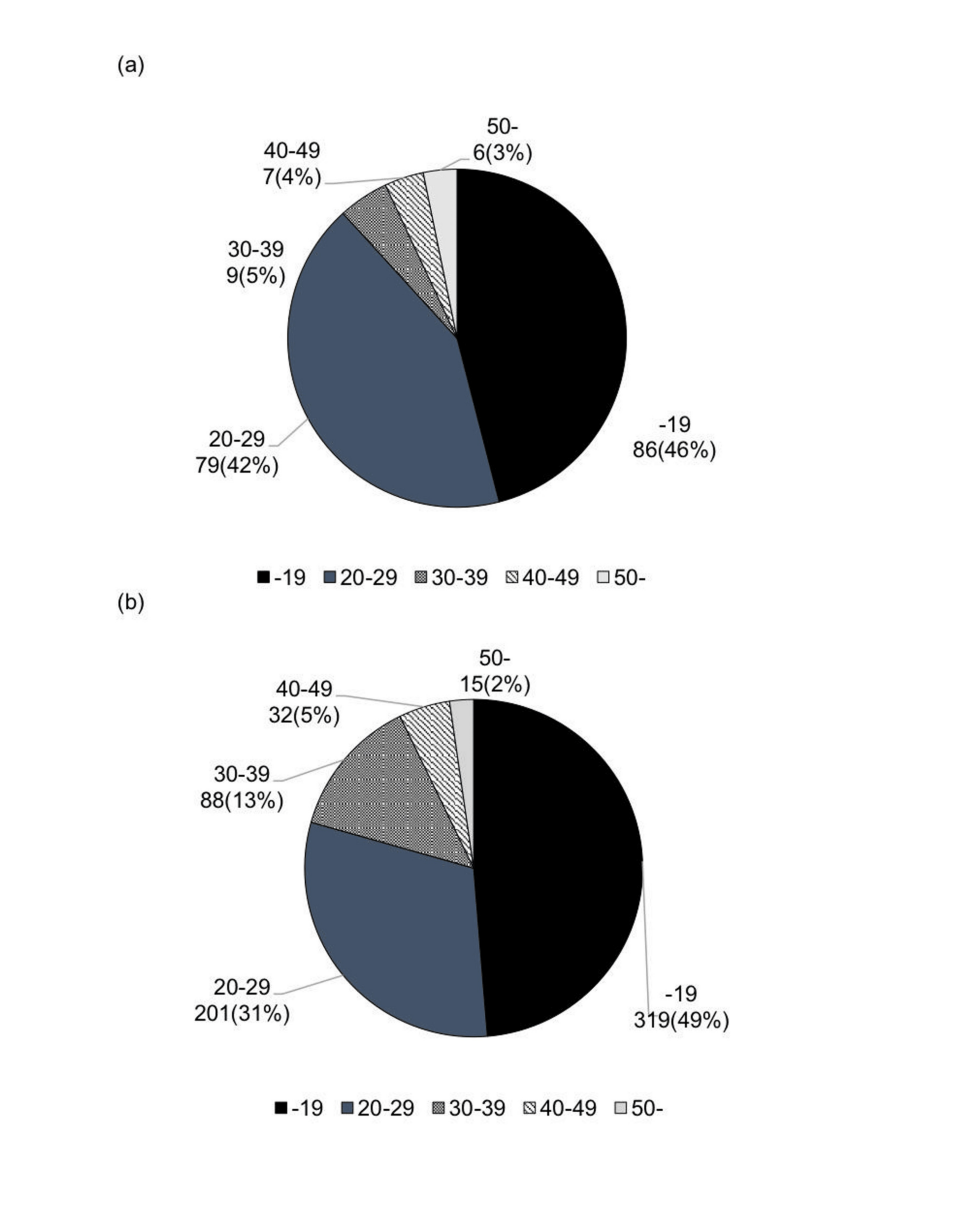
Age distribution of patients using anchor screws and plates (a) First report; (b) current report Author Credits: Mizuki Nakano

A total of 655 patients were included, consisting of 197 males (30%) and 458 females (70%). In the first report [11], there were 37 males (20%) and 150 females (80%), with a similarly high proportion of female patients. In the present study, the age distribution showed that most patients were in their teens (49%) and 20s (31%), with an average age of 23 ± 10 years. Compared to the first report, the proportion of patients in their 30s increased from 5% to 13% (Figure 1).

**Figure 2:**
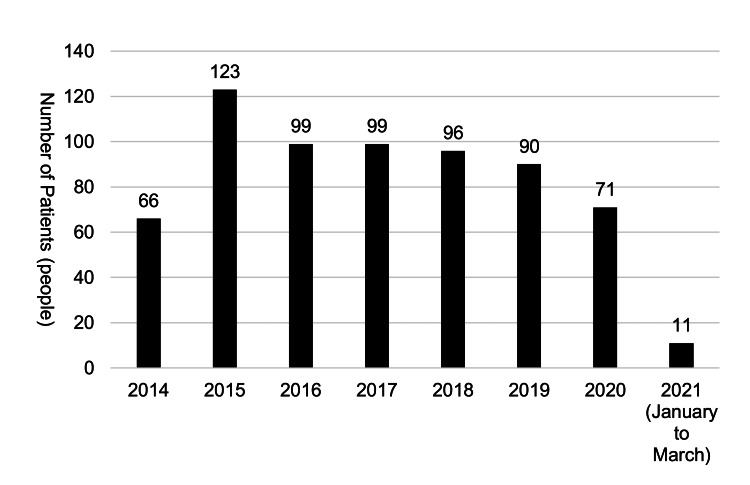
Annual trend in the number of patients using anchor screws and plates Author Credits: Mizuki Nakano

In terms of yearly trends, a decrease in cases was observed starting around 2020 (Figure 2).

Annual trends in the breakdown of self-funded and public medical insurance-covered treatments for patients using anchor screws and plates

**Figure 3:**
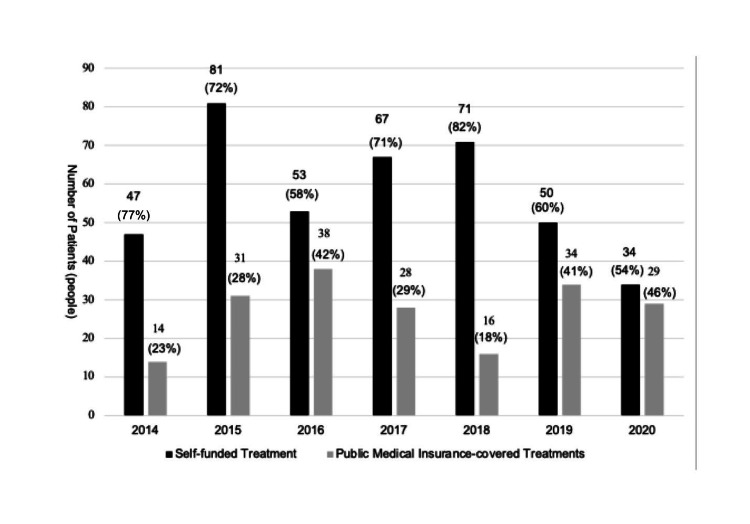
Annual trend in the breakdown of self-funded and public medical insurance-covered treatments for patients using anchor screws and plates Author Credits: Mizuki Nakano

Among the treatment types, 409 patients (62%) underwent self-funded implant treatments, whereas 246 patients (38%) received implants for public medical insurance-covered conditions, with 193 (79%) patients presenting with jaw deformities (Figure 3).

In 2014, the initial year of introducing anchor screws into public medical insurance-covered treatment, the proportion of public medical insurance-covered cases was 23%, which increased to 46% by 2020, indicating a rising trend in public medical insurance-covered treatments.

Malocclusion type according to Angle's classification in patients using anchor screws

**Figure 4:**
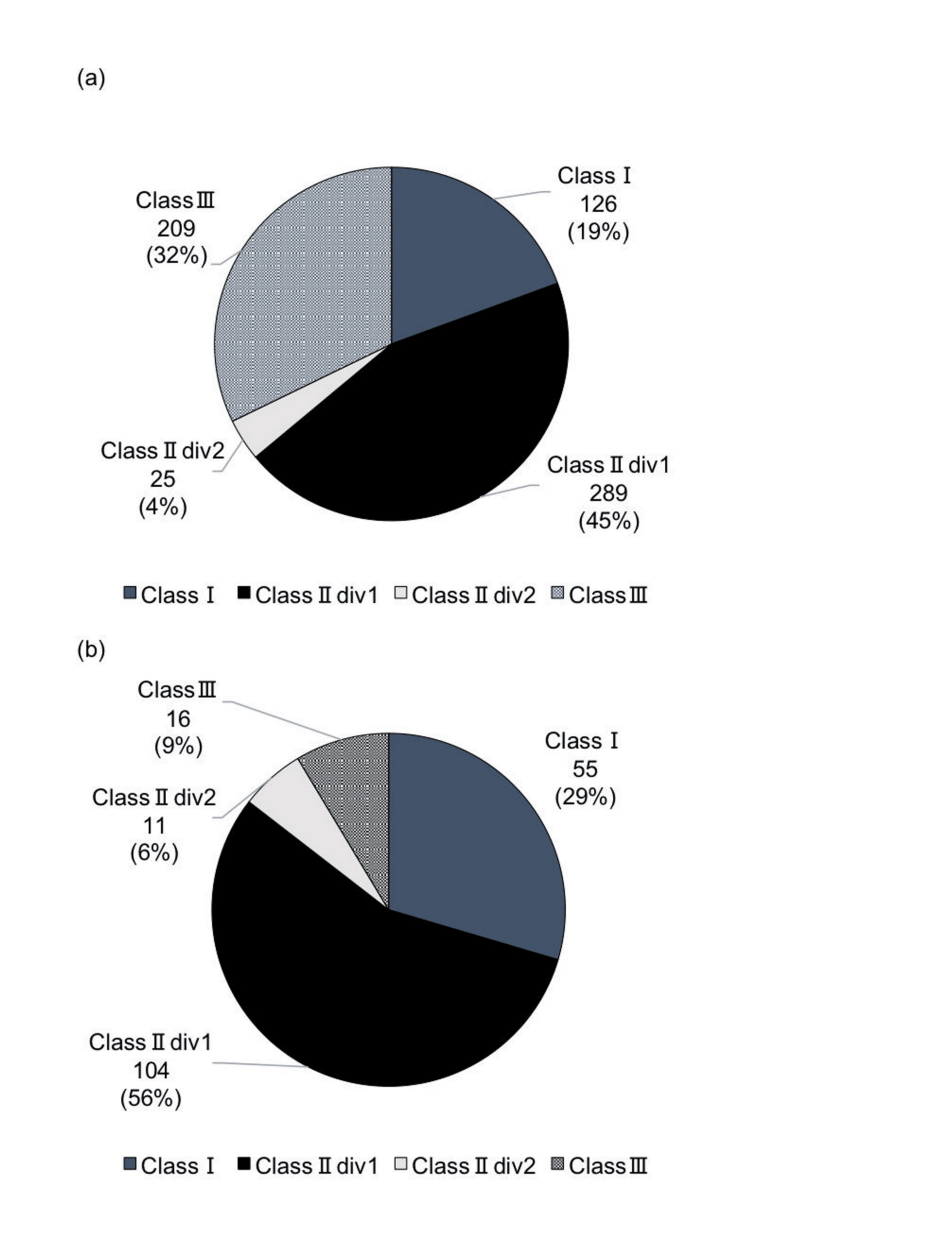
Breakdown of Angle's malocclusion classification among patients receiving anchor screws (a) First survey; (b) current survey Author Credits: Mizuki Nakano

Based on Angle's classification, the distribution was as follows: Class II, Division 1 accounted for 289 (45%), Class III for 209 (32%), Class I for 126 (19%), and Class II, Division 2 for 25 (4%) (Figure 4).

Additionally, 494 cases (75%) involved extractions, and 161 cases (25%) did not involve extractions. In the first report [11], the proportion of Angle Class III patients was 9%, which increased to 32% in the current report.

Purpose of anchor screw use in the maxilla and mandible

**Figure 5:**
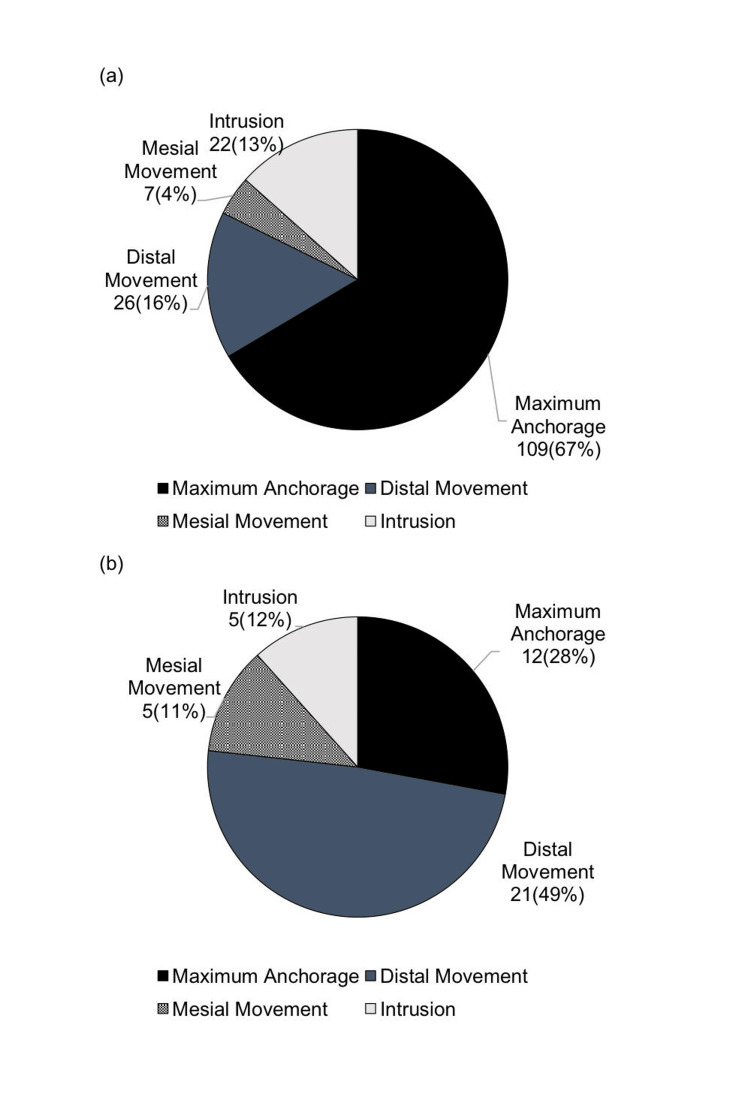
Purpose of anchor screw use (first survey) (a) Maxilla; (b) mandible Author Credits: Mizuki Nakano

**Figure 6:**
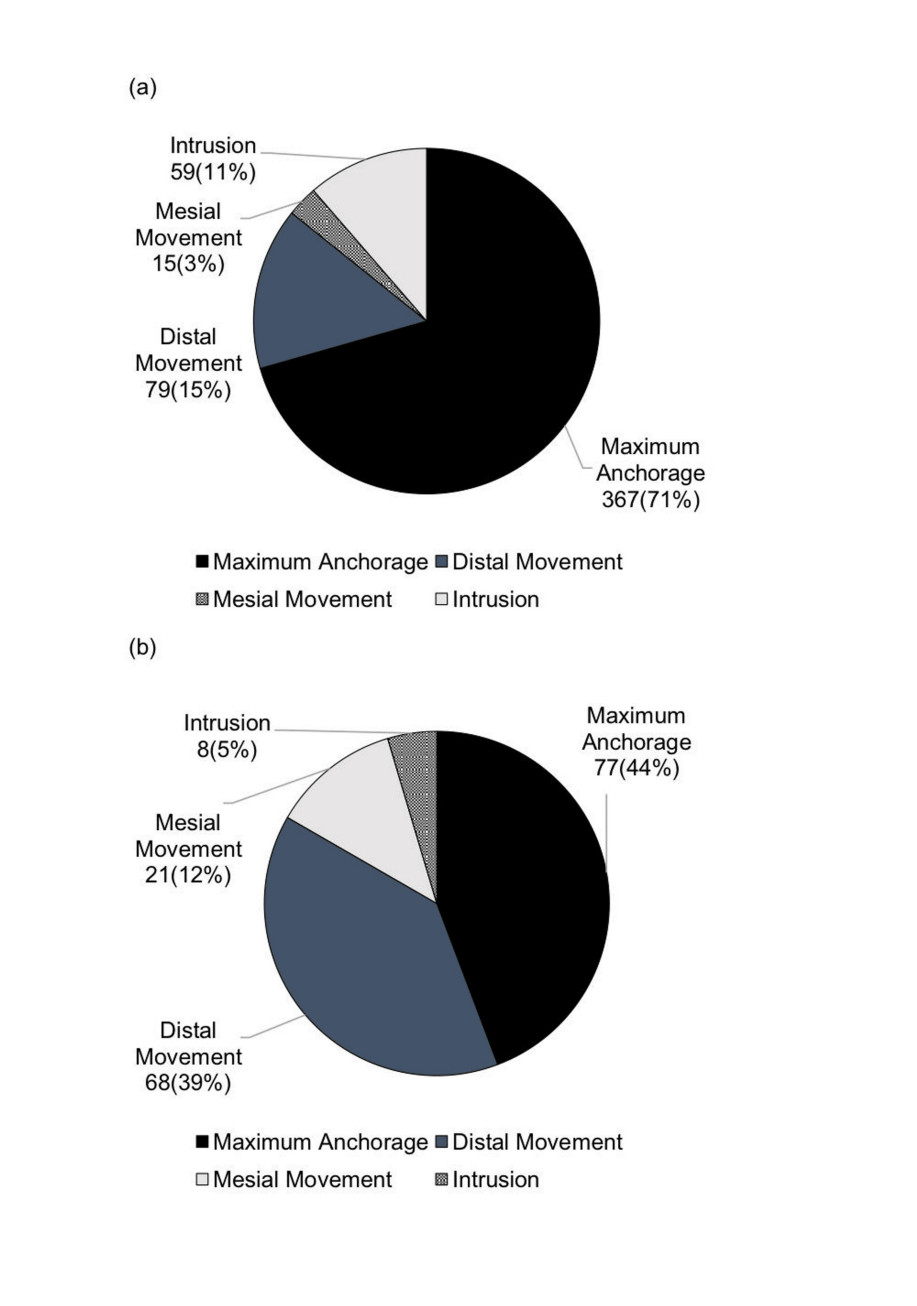
Purpose of anchor screw use (current survey) (a) Maxilla; (b) mandible Author Credits: Mizuki Nakano

In the current report, the primary purpose of use in the maxilla was maximum anchorage, accounting for 367 (71%), followed by distal movement at 79 (15%). In the mandible, maximum anchorage accounted for 77 (44%) and distal movement for 68 (39%). Maximum anchorage was the most commonly used in both jaws. In the current report, the proportion of maximum anchorage increased significantly from 12 (28%) to 77 (44%) (Figures 5, 6)

Comparison of anchor screw usage purposes in patients with jaw deformities (193 patients) and patients with cleft lip and palate (27 patients)

In the posterior maxilla, the primary purpose for patients with jaw deformities was maximum anchorage 101 (65%), followed by distal movement 18 (12%) and intrusion 17 (11%). For patients with cleft lip and palate, the primary purpose was also maximum anchorage in nine (60%) patients, followed by intrusion in three (20%) and mesial movement (7%).

**Figure 7:**
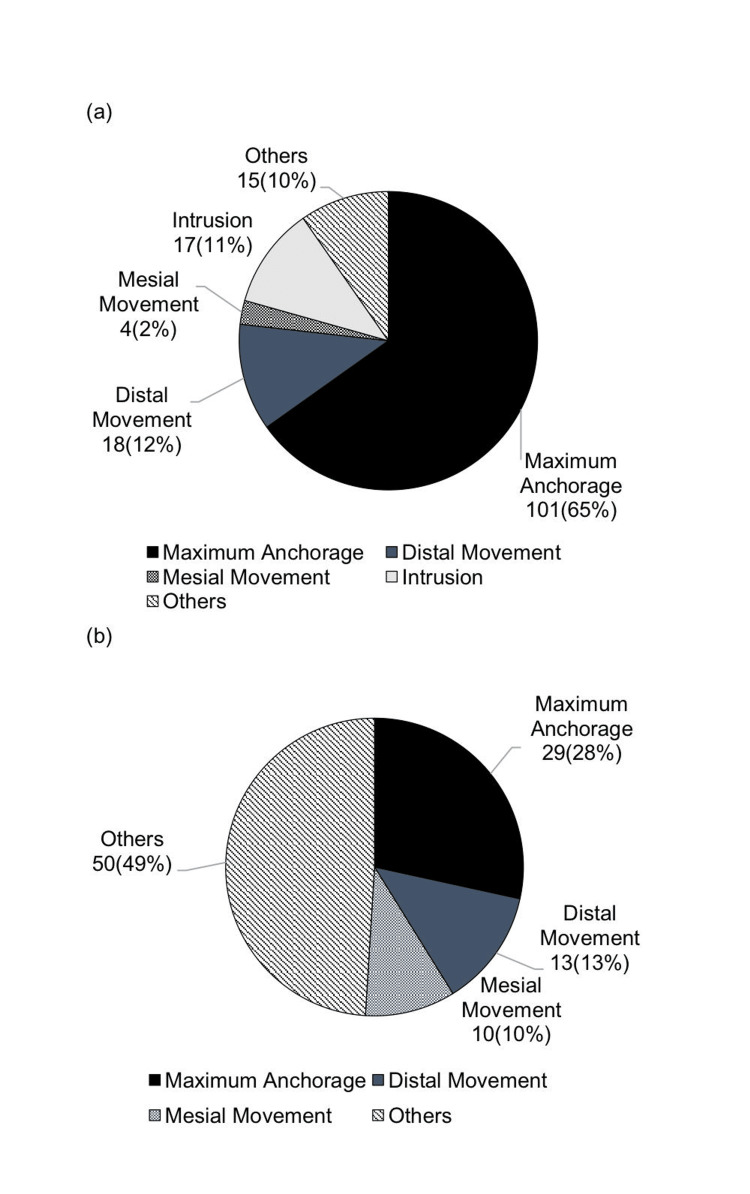
Breakdown of anchor screw usage purposes in patients with jaw deformities (a) Posterior maxilla; (b) posterior mandible Author Credits: Mizuki Nakano

**Figure 8:**
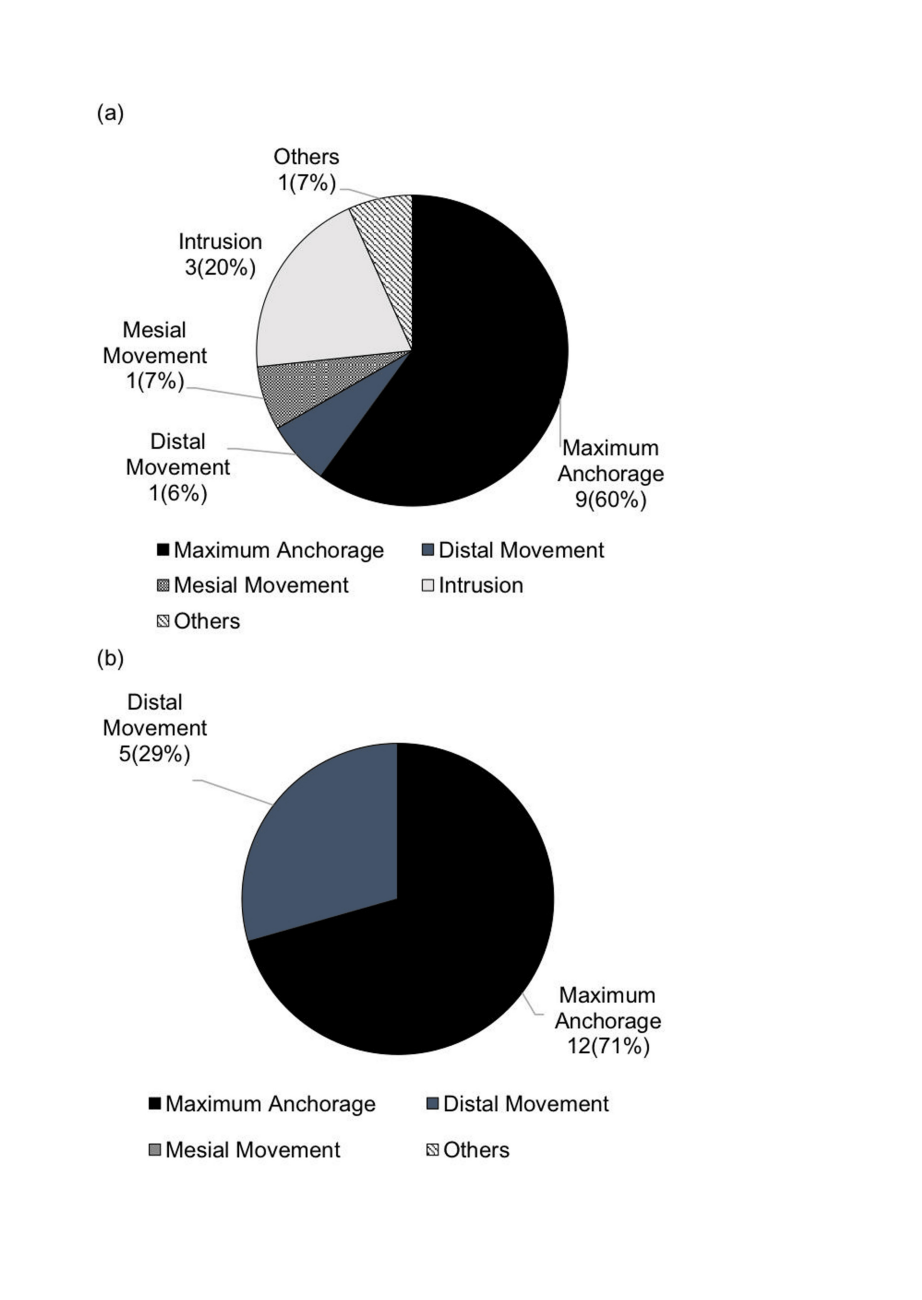
Breakdown of anchor screw usage purposes in patients with cleft lip and palate (a) Posterior maxilla; (b) posterior mandible Author Credits: Mizuki Nakano

In the posterior mandible, for patients with jaw deformities, maximum anchorage accounted for 28%, distal movement for 13%, and mesial movement for 10% (Figure 7). For patients with cleft lip and palate, maximum anchorage accounted for 12 (71%) and distal movement for five (29%) (Figure 8).

Breakdown of the types of anchor screws and plates used

**Figure 9:**
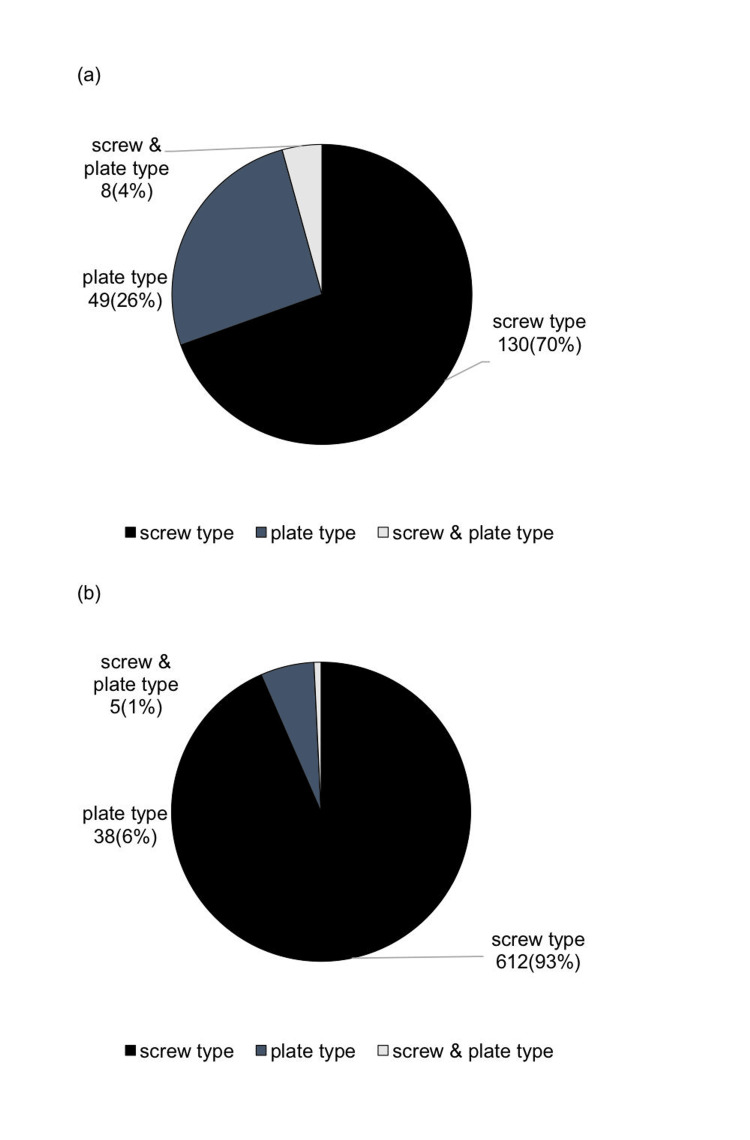
Breakdown of types of anchor screws and plates used (a) First survey; (b) current survey Author Credits: Mizuki Nakano

Among the types of anchor screws used, screw-type anchor screws accounted for 612 cases (93%), plate-type for 38 cases (6%), and a combination of both types for five cases (1%) (Figure 9).

In the first report [11], the proportion of screw-type anchor screws was 130 (70%), which increased to 612 (93%) in the current report.

Relationship between Angle's classification and purpose of anchor screw use

Regarding the purpose of anchor screw use, a chi-square test was performed on the posterior maxilla, and a significant difference was found at P=0.000, so a residual analysis was performed. The results showed that maximum anchorage was the most frequent in Class Ⅱ, Division 1, followed by distal movement. In Class III cases, the frequency of maximum anchorage was lowest, with distal movement also being less frequent. The chi-square test was also performed on the posterior mandible, and a significant difference was found at P=0.000, so a residual analysis was performed. In the posterior mandible, Class II, Division 1 cases had the lowest frequency of distal movement, as well as a lower frequency of maximum anchorage. For Class III cases, distal movement had the highest frequency, followed by maximum anchorage (Tables 1, 2).

**Table 1:** Breakdown of purposes of anchor screw use in the posterior maxilla based on Angle's classification Angle's classification is a classification of the occlusion of the maxilla and mandible according to their mesiodistal relationship. There are four main classifications (Class Ⅰ, Class Ⅱ Division 1, Class Ⅱ Division 2, and Class Ⅲ). A chi-square test was conducted at a significance level of 5% (P<0.05). Author Credits: Mizuki Nakano

Posterior Maxilla (No. of Patients)		Patients	%	Adjusted Residual
Class Ⅰ (100)	Maximum Anchorage	79	79	1.8
	Distal Movement	11	11	-1.3
	Mesial Movement	3	3	0.2
	Intrusion	7	7	0.4
	Others	0	0	-1
Class II Division 1 (261)	Maximum Anchorage	197	68.2	5.8
	Distal Movement	51	17.6	3.9
	Mesial Movement	4	1.4	-1.2
	Intrusion	8	2.8	-2.2
	Others	1	0.3	-0.8
Class II Division 2 (19)	Maximum Anchorage	12	63.2	-0.8
	Distal Movement	6	31.6	1.9
	Mesial Movement	0	0	-0.8
	Intrusion	1	5.2	-0.2
	Others	0	0	-0.4
Class Ⅲ (110)	Maximum Anchorage	76	69.1	-6.9
	Distal Movement	10	9.1	-3.9
	Mesial Movement	5	4.5	0.3
	Intrusion	16	14.5	2.2
	Others	3	2.7	1.8

**Table 2:** Breakdown of purposes of anchor screw use in the posterior mandible based on Angle's classification Angle's classification is a classification of the occlusion of the maxilla and mandible according to their mesiodistal relationship. There are four main classifications (Class Ⅰ, Class Ⅱ Division 1, Class Ⅱ Division 2, and Class Ⅲ). A chi-square test was conducted at a significance level of 5% (P<0.05). Author Credits: Mizuki Nakano

Posterior Mandible (No. of Patients)		Patients	%	Adjusted Residual
Class Ⅰ (43)	Maximum Anchorage	25	58	3
	Distal Movement	6	14	-2.3
	Mesial Movement	3	7	-0.6
	Intrusion	6	14	2.5
	Others	3	7	1.9
Class Ⅱ Division 1 (29)	Maximum Anchorage	14	48.3	-5
	Distal Movement	7	24.1	-5.9
	Mesial Movement	6	20.8	-1.5
	Intrusion	1	3.4	-2.7
	Others	1	3.4	-1.4
Class Ⅱ Division 2 (4)	Maximum Anchorage	2	50	-0.6
	Distal Movement	0	0	-1.7
	Mesial Movement	0	0	-0.9
	Intrusion	2	50	2.2
	Others	0	0	-0.5
Class Ⅲ (108)	Maximum Anchorage	38	18.1	3.3
	Distal Movement	55	26.2	9.1
	Mesial Movement	10	4.8	1.6
	Intrusion	4	1.9	-0.1
	Others	1	0.5	-0.8

## Discussion

Trends in the use of anchor screws and plates for orthodontic treatment in our department

In 1997, our department began using dental implants as a source of anchorage for orthodontic treatment [13]. In 2010, ethical approval was obtained, and our department established consent forms and clinical pathways, as well as conducting annual training for oral surgery and orthodontics staff. Influenced by the 2012 pharmaceutical approval and the establishment of guidelines by the Japan Orthodontic Society [14], we have established orthodontic treatment using bone anchorage as a predictable treatment mechanic. Furthermore, in 2014, anchor screws began to be covered by insurance for orthodontic treatment in patients with jaw deformities and congenital conditions. In the first report [11], 187 patients received anchor screws and plates in the 10 years from 2004 to 2013. However, in the current report, covering a seven-year period from 2014 to 2021, 655 patients were treated, confirming an increase in the application of anchor screws and plates in orthodontic treatment. This result suggests that since anchor screws and plates have become applicable in orthodontic treatment, the mechanics have become more widely accepted among clinicians and applied more frequently. Additionally, the introduction of public medical insurance coverage for anchor screws in certain conditions likely contributed to this increase.

The male-to-female ratio among patients using anchor screws and plates in this study was 1:2, whereas in the first report [11], it was 1:4, indicating an increase in the proportion of male patients. According to Nagai et al. [15] (1990), the male-to-female ratio among adult patients over 19 years old in our hospital was 1:2. In Kase et al.'s report [16] (2013), females accounted for 60% of all patients, and in Iwamoto et al.'s report [17] (2014), the figure was 64.8%. Additionally, Nagai et al. [15] reported that malocclusions such as maxillary protrusion, crowding, and open bite, which are more commonly seen in females, are frequently treated with anchor screws. Reports from our hospital and other institutions [18-21] indicate an increasing trend in orthodontic public medical insurance coverage for patients with jaw deformities and congenital conditions. The current report showed an increase in the proportion of male patients compared to the first report, which may be attributed to the addition of patients eligible for public medical insurance-covered orthodontic treatment, including those with jaw deformities and congenital conditions.

In the current report, the proportion of patients in their 30s also increased compared to the first report [11]. This suggests an increase in comprehensive treatment needs due to more extensive intraoral issues in adult orthodontic patients.

Malocclusion type according to Angle's classification in patients using anchor screws

In the breakdown of anchor screw applications, the molar relationship classifications were 45% for Angle Class II, Division 1, and 32% for Class III. Similar to our findings, Kawasaki et al. [22] reported that maxillary protrusion was the most common outcome.

Additionally, compared to the first report [11], there was a significant increase in the application of anchor screws for Class III patients. In the current report, cross-tabulation showed that maximum anchorage was the most common purpose (69.1%) for the maxillary dentition in Class III cases, which may be attributed to an increase in surgical orthodontic treatment applications for skeletal mandibular prognathism.

Comparison of anchor screw usage purposes in patients with jaw deformities and patients with cleft lip and palate

In the maxillary molar region, for patients with jaw deformities, maximum anchorage was commonly used, followed by distal movement, while maximum anchorage was the most frequent purpose for patients with cleft lip and palate. In the mandibular molar region, maximum anchorage was frequently used for patients with jaw deformities, followed by distal movement. For patients with cleft lip and palate, distal movement was also the most common purpose following maximum anchorage.

Sasaki et al. [23,24] suggested that distal movement and intrusion of molars using anchor screws could lead to an increase in non-extraction treatments and enable vertical control of molars during preoperative orthodontic treatment, potentially resulting in more cases where less invasive surgical methods are chosen.

In this study, the sample size of cleft lip and palate patients was small, indicating a need for a longer-term study. There are reports [25] that found no significant differences in success rates between cleft lip and palate patients and non-cleft patients based on factors such as placement site, age, gender, screw diameter, and length, suggesting the potential for expanded applicability in the future.

Breakdown of the types of anchor screws and plates used

Approximately 93% of cases used screw-type anchor screws. Factors influencing the choice of orthodontic anchor screw types include anatomical structures such as the maxillary sinus floor, mandibular canal, and mental foramen position, as well as inter-root distance, cost-effectiveness, and intended use. Screw-type anchors are more frequently used compared to plate types due to their minimally invasive nature, ease of placement and removal, and lower cost. Compared to the first report [11], the proportion of screws used has increased, which may be related to the introduction of public medical insurance coverage for anchor screws in 2014.

Relationship between Angle's classification and purpose of anchor screw use

In the classification of malocclusion according to Angle in patients using anchor screws, Class III cases accounted for 9% in the first report [11], but this increased significantly to 32% in the current report.

For Class III cases, anchor screws are used for either surgical orthodontic treatment or orthodontic treatment alone. In surgical orthodontic treatment, it is expected that they are often used for maximum anchorage in the maxillary molar region. When used solely for orthodontic treatment, they are frequently applied for distal movement in the mandibular molar region. Across the entire maxillary and mandibular arches, the frequency of distal movement is higher in the mandibular arch. However, when looking at Class III cases only, maximum anchorage in the maxillary molar region is the most common purpose (69.1%), suggesting a high usage for surgical orthodontic treatment for skeletal mandibular prognathism.

In Class III cases undergoing surgical orthodontic treatment, the use of anchor screws for maximum anchorage or distal movement in the maxillary molar region offers the advantage of achieving non-extraction treatment and potentially downgrading the surgical approach from bimaxillary surgery to mandibular surgery alone. The current report suggests an increase in such applications compared to the first report [11].

With the rising number of conditions covered by public medical insurance coverage for orthodontic treatment and the increase in orthodontic patients over 30 years old, comprehensive dental care is becoming more essential. Furthermore, improvements in the materials of anchor screws and plates, as well as the maturation of mechanics, are expected to continue expanding their use and applications in the future.

Study limitations

This study has several limitations. Individual data on gender, age, and yearly trends could not be obtained for anchor screws or plates because they were not surveyed separately. The width and properties of the cortical bone also affect the anchor screw anchorage and tooth movement. Since bone properties vary by age and gender, itemizing may change the results. Additionally, malocclusion classification was based solely on Angle's classification, which defines mesiodistal malocclusion and does not focus on vertical malocclusions such as deep bite or open bite. Not focusing on malocclusion, including anterior occlusion, is one of the limitations of this study. It is necessary to monitor clinical statistics for each specific item in the future.

## Conclusions

This study investigated the usage status of anchor screws and plates over a seven-year period from 2014 to 2021 in the Department of Orthodontics at Tokyo Dental College Chiba Dental Center, indicating an increase in the number of Angle Class III patients receiving anchor screws and plates. Notably, the frequency of maximum anchorage usage in the maxillary molar region for surgical orthodontic treatment of skeletal mandibular prognathism increased compared to the first report.

Since the first report, which covered the period up to 2013, there has been an overall increase in the number of cases, and public medical insurance coverage has been expanded to include jaw deformities and congenital abnormalities. This suggests an expansion in the application of anchor screws for public medical insurance-covered conditions, as indicated by the increased frequency of maximum anchorage usage in the maxillary molar region for Class III cases. The clinical application of anchor screws is expected to continue expanding, and it is considered necessary to keep monitoring clinical statistics.
